# A nanoparticle-based immobilization assay for prion-kinetics study

**DOI:** 10.1186/1477-3155-4-8

**Published:** 2006-08-17

**Authors:** Gilles K Kouassi, Joseph Irudayaraj

**Affiliations:** 1 Agricultural and Biological Engineering, The Pennsylvania State University, State College, University Park, PA 16802, US; 2 Bindley Biosciences Center, Purdue University, 225 S. University St., West Lafayette, Indiana 47907, US

## Abstract

Magnetic and gold coated magnetic nanoparticles were synthesized by co-precipitation of ferrous and ferric chlorides, and by the micromicelles method, respectively. Synthesized nanoparticles were functionalized to bear carboxyl and amino acid moieties and used as prion protein carriers after carbodiimide activation in the presence of *N*-hydroxysuccinimide. The binding of human recombinant prion protein (huPrPrec) to the surface of these nanoparticles was confirmed by FTIR and the size and structures of the particles were characterized by transmission electron microscopy. Findings indicate that the rate of prion binding increased only slightly when the concentration of prion in the reaction medium was increased. Rate constants of binding were very similar on Fe_3_O_4_@Au and Fe_3_O_4_-LAA when the concentrations of protein were 1, 2, 1.5, 2.25 and 3.57 μg/ml. For a 5 μg/ml concentration of huPrPrec the binding rate constant was higher for the Fe_3_O_4_-LAA particles. This study paves the way towards the formation of prion protein complexes onto a 3-dimensional structure that could reveal obscure physiological and pathological structure and prion protein kinetics.

## Background

Prion diseases also called Transmissible Spongiform Encephalopathies (TSE) are a group of degenerative diseases that feature the pronounced accumulation, in certain brain regions, of a misfolded isomer PrP^sc ^of the cellular prion protein (PrP^c^) [1-3]. Spongiform encephalopathy in cattle, scrapie in sheep, Gerstmann-Straussler Scheinker in human, and Creutzfied-Jacob disease are caused due to the misfolding of protein denoted as prion protein [1-4]. Understanding the basis of prion disease revolves around understanding how the normal protein, PrP^c ^is converted into its abnormal form, PrP^sc^. Hypothesis suggest that prion infection is associated with a conformational transition between the two forms [1-5]. In a broader sense, prions are elements that impart and propagate variability through a multitude of conformations of normal cellular proteins [6]. However, the mechanism by which this conversion occurs is not clearly known. Cui *et al*. [6] and Spencer *et al *[7] proposed that this conversion involves a switch in the conformation from a structure rich in α-helix to the one rich in β-sheet through a spatial arrangement and molecule folding.

Protein unfolding is associated with the disruption of interactions leading to a loss in the secondary structure (the fold of α-helices, β-strands and turns, and tertiary structure – the packing of the secondary structural elements and the amino acid side chains [8]). In the last two decades a fundamental concern that arose was on the issues of prion related diseases. For example, meat exported from UK has been banned by a number of countries as a precautionary measure against Bovine Spongiform Encephalopathy (BSE) after the major outbreak of mad cow disease in 1996. Although the cause of the infection remains unknown, it was suggested that meat and bone meal imported from other countries may have been incorporated into feed supplements before these countries could adopt a suitable feed control strategy [9]. In 2003, BSE was detected in one cow in Canada and another in the U.S which lead to the killing of more than 2700 animals and subsequent testing to trace the history and source of contamination [10]. Adequate methods to detect and screen prion related diseases could prevent the mass killing of animals while simultaneously ensuring the safety of animal-based food products. Since identification and quantification of proteins and their folding mechanism are very important in disease diagnosis, nanotechnology based approaches could perhaps be used to develop detection assays that are very sensitive with ability to differentiate between structural elements [11].

It is well known that intermolecular covalent cross-linking of functional groups in proteins has proved to be a very useful approach in the study of structure-function relationship in proteins [12]. Furthermore, insights into protein folding could be better evaluated and the molecule manipulated via appropriate bioconjugation strategies using nanotechnology based approaches. Nanostructures could thus be construed as a natural choice. The structure-function relationship of proteins or its stability depends on the combination of several properties which have to be fulfilled by the amino acid at a certain position in the protein. For example, a relationship between the stability of the hydrophobic moieties and the buried residues with conformational stability has been found [13]. This suggests that the conformation which involves the 3-dimensional arrangement of the protein molecule affects its functionality. Examination of the 3-D structure allows the protein to exhibit conformations that may reveal details of its structure and help understand its activity [13]. Conformational changes occur via distinct molecular domains, as defined by their binding to monoclonal antibody fragments. However, a flat 2-D surface offers less binding capacity than a 3-D structure, thus increasing considerably the sensitivity of measurement [15]. Moreover, the 3-D structure yields superior signal over a flat substrate and enhances the quantity of adsorbed protein per unit area [16]. In this context, metal nanoparticles could serve as potential carriers and/or anchor materials for biomolecules. Magnetic nanoparticles for example have been reported as support structures for biological materials including proteins, peptides, enzymes, DNA, because of their uniqueness [17-19]. Magnetically labeled molecules could be directed or driven to a specific location in a biological system using suitable magnetic fields. However, aggregation is a known problem when utilizing magnetic nanoparticles. While magnetic nanoparticles have unique advantages, considerable attention has also been placed on the functionalization of gold nanoparticles because of their excellent biocompatibility and established synthesis protocols. Furthermore, the possible application of thiol chemistry on gold surface allows the attachment of molecules with relative ease using various thiol linkers [20-23]. Hence, when magnetic nanoparticles are coated with a gold shell, the magnetic character and attributes could be preserved, and all the benefits of gold surfaces could be harnessed in the areas of biosensors and bioseparations when such a biocomplex is functionalized.

In the present study, we report the kinetics of binding of prion protein to magnetic and gold coated magnetic nanoparticles, after modification of the surface chemistries of these materials. The modification of magnetic nanoparticles consists of the chemosorption of L-aspartic acid (LAA), while gold coated magnetic nanoparticles were carboxylated using mercaptopropionic acid. The binding of prion protein to the particles was achieved directly after the activation of carbodiimide in the presence of *N*-hydroxysuccinimide. The change in the rate of binding in response to the variation of the protein density in the reaction medium was also examined.

## Results and discussion

**Figure 1:**
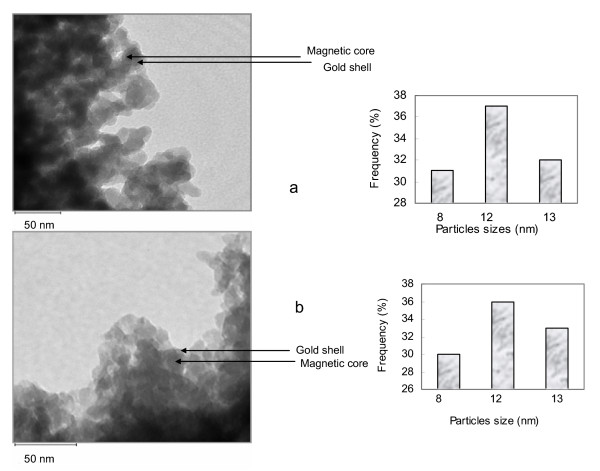
TEM images and particle sizes distribution of gold coated magnetic nanoparticles- Fe_3_O_4_@Au (a) and huPrPrec functionalized gold coated magnetic nanoparticles- Fe_3_O_4_@Au-huPrPrec (b).

**Figure 2:**
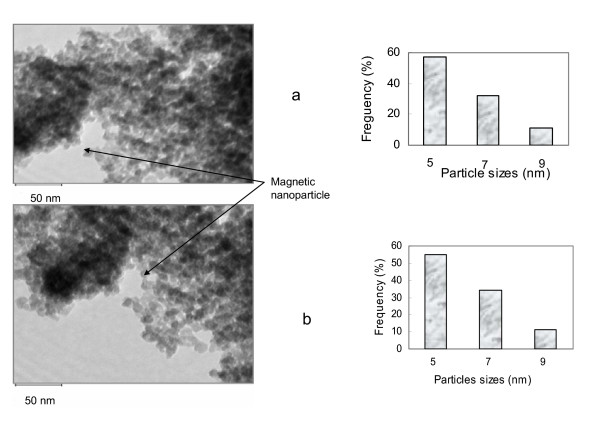
TEM images and particle sizes distribution of magnetic nanoparticles- Fe_3_O_4_-LAA (a) and huPrPrec functionalized magnetic nanoparticles Fe_3_O_4_-LAA- huPrPrec (b).

**Figure 3:**
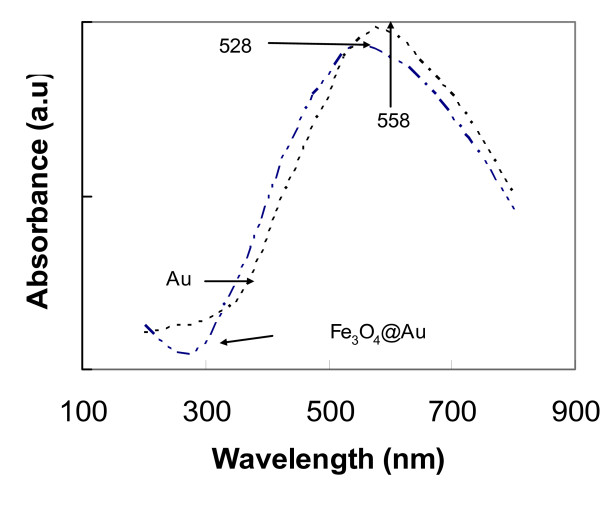
Optical properties of pure gold solution and gold coated magnetic nanoparticles. The absorption maxima were found at 520 and 558 nm for Au and Fe3O4@Au, respectively.

The particles generally have a spherical shape and span a distribution of particle sizes. The Fe_3_O_4_@Au nanoparticles were bigger and are in the range between 8 and 13 nm while Fe_3_O_4_-LAA were between 5 and 9 nm. The difference in size is attributable to the gold coating, assuming that the gold shell around the magnetic particles contributes to an increase in size. TEM images of Fe_3_O_4_@Au nanoparticles (a) and huPrPrec functionalized gold coated magnetic nanoparticles (Fe_3_O_4_@Au-huPrPrec) (b) are shown in Figure 1, and Fe_3_O_4_-LAA nanoparticles (a) and huPrPrec functionalized magnetic nanoparticles – Fe_3_O_4_-LAA-huPrPrec (b) are shown in Figure 2. The presence of the gold shell around the magnetic nanoparticles was confirmed by the difference in absorption spectra of pure gold and Fe_3_O_4_@Au colloid prepared in the same way. The absorption band of the gold colloid was noted to have its maximum at 528 nm, while the Fe_3_O_4_@Au colloid exhibited a maximum at 558 nm (Figure 3). Results obtained were consistent with that reported by Jun et al. [22] and Rivas et al. [24] for the absorption maximum (526 nm) of pure gold colloid, while the absorption maximum for the Fe_3_O_4_@Au was consistent with the value reported by Jun et al. [22].

**Figure 4:**
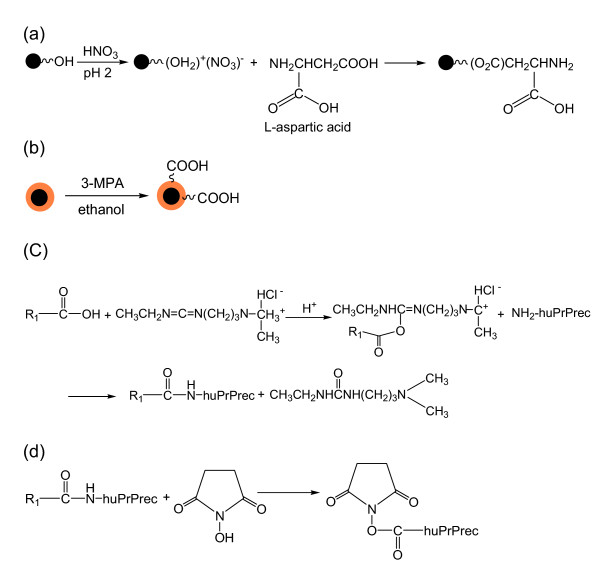
Functionalization of magnetic and gold coated magnetic nanoparticles: the chemisorption of LAA onto Fe_3_O_4 _(a), the carboxylation of Fe_3_O_4_@Au using mercaptopropionic acid (b), the activation of the carboxyl groups on the particle, the formation of *N*-hydroxysuccinimidyl ester in the presence of EDC (c), and the immobilization of huPrPrec onto particles (d). R_1 _denotes nanoparticles.

The incorporation of carboxyl groups onto the Fe_3_O_4_@Au particles consisted of placing the nanoparticles for two nights in an ethanolic solution of 3-MPA to allow binding to occur between the gold surface and the thiol group via the well known thiol chemistry. For the functionalization of magnetic nanoparticles, LAA was chemisorbed [23] onto the particle surface to provide a particle surface with carboxyl and amino groups. These groups were further activated by carbodiimide for the immobilization of prion protein. Figure 4 describes the chemisorption of LAA onto Fe_3_O_4 _(a), the carboxylation of Fe_3_O_4_@Au using mercaptopropionic acid (b) and the immobilization of prion protein onto the particles using the carbodiimide bridge. Briefly, the magnetic nanoparticles were activated with nitric acid to favor the attachment of L-aspartic acid bearing carboxyl groups onto the magnetic nanoparticles. The formation of amide bonds between carboxylic acids and amines was catalyzed by carbodiimide which activates the carboxyl groups on the linkers to form O-*urea *derivatives. Succinctly, addition of *N*-hydroxysuccinimide catalyzed the formation of the intermediate active esters that further reacts with the amine function of the prion protein to yield the amide bond between the protein and the carboxyl groups on the particles.

### FTIR

**Figure 5:**
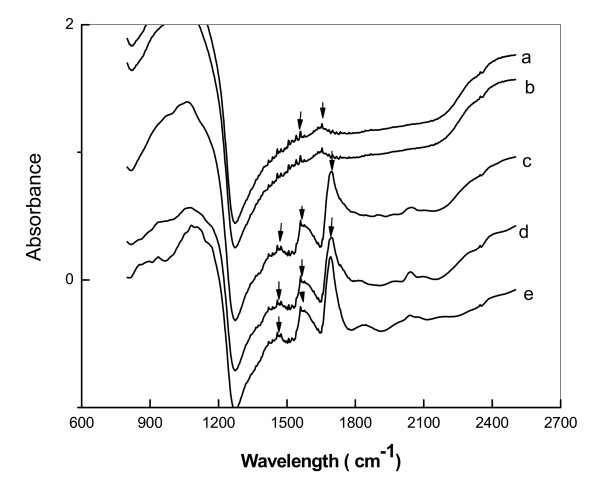
FTIR spectra of Fe_3_O_4_@Au (a), Fe_3_O_4_-LAA (b) huPrPrec (c), Fe_3_O_4_@Au-huPrPrec (d) Fe_3_O_4_-LAA-huPrPrec(e).

The adsorption of monolayers and biofunctionalization of nanoparticles were qualitatively assessed by FTIR spectroscopy. Figure 5 shows the FTIR spectra of Fe_3_O_4_@Au, Fe_3_O_4_-LAA, huPrPrec, Fe_3_O_4_@Au-huPrPrec, and Fe_3_O_4_-LAA-huPrPrec. The coating of Fe_3_O_4_@Au and Fe_3_O_4 _with carboxyl groups and LAA was noted by the appearance of various peaks in the regions from 1400 to 1650 cm^-1 ^in the spectra of Figure 5a and 5b. The multitude of small peaks crowding the spectra with features associated with various functional groups hinder identification of peaks specific to carbonyl and amine groups in the chemisorbed regions making the differentiation between Fe_3_O_4_@AuCOOH (Fe_3_O_4_@Au bearing carboxyl groups) and Fe_3_O_4_-LAA difficult. Spectra in Figure 5c depicts the functional groups related to pure huPrPrec via characteristic bands of proteins at 1490, 1541, and 1645 cm^-1 ^wave numbers assignable to the symmetric stretching of the dissociated carboxylic group originating from the amino acid, amide I and amide II as shown in the spectra of Figure 5d and 5e. In the 1415-1300 cm^-1 ^region of the spectra in Figure 5c, d and 5e a weak band typical to the spectra of the carboxylate group attributable to proteins was also noted. The presence of protein characteristics on the spectra of pure huPrPrec and on the spectra of Fe_3_O_4_@AuCOOH-huPrPrec, Fe_3_O_4_-LAA-huPrPrec confirmed that huPrPrec was effectively bound to the nanoparticles.

### Binding kinetics

**Figure 6:**
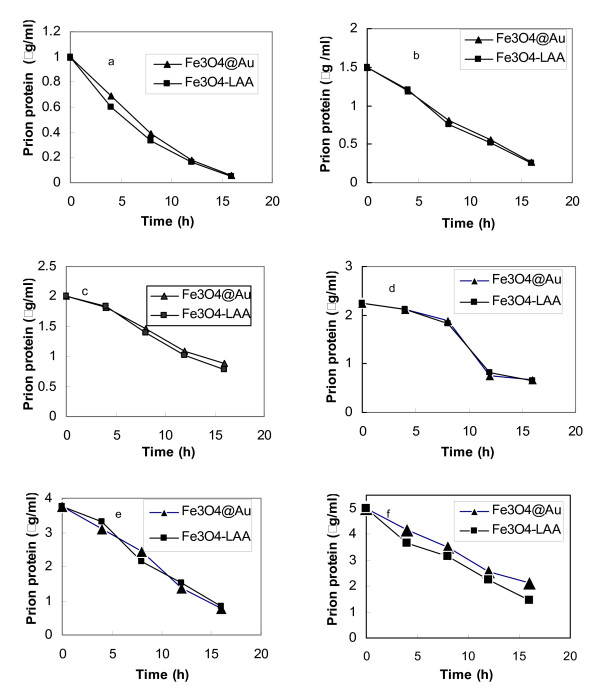
Plots of huPrPrec binding onto Fe_3_O_4_@Au and Fe_3_O_4_-LAA (a) as a function of time for various concentrations of huPrPrec.

**Figure 7:**
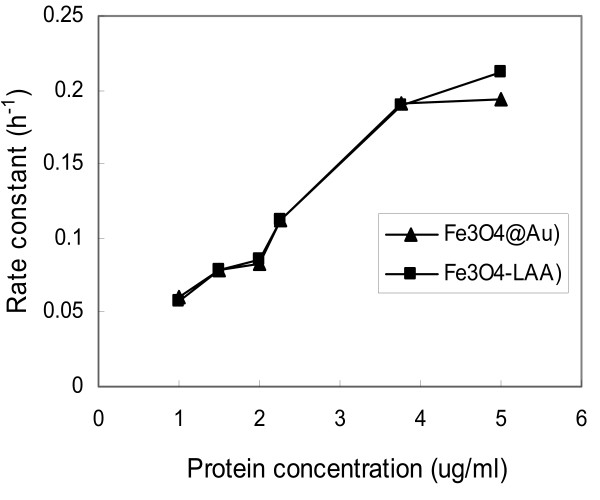
Dependence of the rate constants of huPrPrec binding on the concentrations of reacting huPrPrec.

huPrPrec was immobilized onto functionalized Fe_3_O_4_@Au and Fe_3_O_4_-LAA particles after activation by EDC in the presence of NHS. The amount of huPrPrec was determined spectrophotometrically using different concentrations of huPrPrec. The rate of binding expressed as the change in the concentration of unbound protein in the reaction medium was measured using a linear regression analysis of the plots of huPrPrec concentration as a function of time. The coefficient of variation between replicates was less than 4%. Figure 6a, b, c, d, e and 6f show plots of the decrease in huPrPrec when the initial concentrations were 1, 1.5, 2, 2.25, 3.75, and 5 μg/ml, respectively. Rate constants of huPrPrec presented in table 1 shows an increase in the initial concentration of prion protein from approximately 0.06 to 0.194 h^-1^, and 0.058 to 0.212 h^-1 ^for Fe_3_O_4_@Au and Fe_3_O_4_-LAA, respectively, in the range of huPrPrec concentrations tested. For each concentration of protein, the rate constant was about the same, irrespective of the type of nanoparticles, except when a concentration of 5 μg/ml of huPrPrec was used. The binding occurred gradually and reached approximately 94%, 87%, 65%, 73%, 79%, and 74% for Fe_3_O_4_@Au and 94%, 87%, 67%, 73%, 81%, and 71% for Fe_3_O_4_-LAA, when the initial concentrations of huPrPrec were 1.5, 2, 2.25, 3.75, and 5 μg/ml, respectively, after 20 h of reaction. Figure 7 show plots of the rate constant as a function of the initial concentration of huPrPrec. The trend observed shows that the rate constant increased exponentially with the initial concentration of huPrPrec. Fischer et al. [25] immobilized the disease associated prion protein using 0.1 mg/ml of reacting prion protein for immobilizing the disease associated prion protein solution onto magnetic beads. Here, we were able to quantify down to 0.6 % of 1 μg/ml of reacting huPrPrec. The low concentration of huPrPrec coupled to the increase in the binding rate constants observed with increased protein concentration suggests that the detection methodology is sensitive. Table 2 shows the percentage of huPrPrec binding to the nanoparticles. The trend was quite similar for the immobilization carried out using both carriers although the overall binding rate constants were slightly higher in Fe_3_O_4_-LAA than the Fe_3_O_4_@Au conjugates. This indicated that binding was slightly more effective when Fe_3_O_4_-LAA complex was used. The bigger sizes of Fe_3_O_4_@Au should have favored the rate of binding since it offers a greater surface area, but this is not the case here where Fe_3_O_4_-LAA exhibited a better binding affinity. The difference in the affinity of binding could be attributed to the fact that LAA possess amino acids that originated from the protein that may exhibit higher affinity to the surface of the particles. The difference in the rate constants observed at this concentration may be associated with the difference in functional groups on the surface of the nanoparticles used. Prion protein is a complex entity, and although numerous binding partners have been found for the protein, its function still remains unclear, since each portion of the protein has its own functional properties [26]. Although significant effort has been made in elucidating prion related diseases, numerous questions on the structure and conformation of the protein are still to be answered. Huang et al [16] demonstrated that immobilizing specific proteins onto magnetic nanoparticles allows the molecules to expand and acquire a better conformation that could lead to an improved activity. As the possibility to move magnetic nanoparticles using an external magnetic field is an essential asset for molecular manipulation for diagnosis purposes, the immobilization of prion protein onto magnetic and gold coated magnetic nanoparticles and the kinetics data obtained in this study may contribute to an improved biological assay in which the protein could be directed toward target locations such as monoclonal prion protein antibodies [8] by appropriate directed magnetic fields. Furthermore, data on binding kinetics can be useful in the design and evaluation of molecular carriers and in the measurement of the efficacy of the surface chemistry developed. Developing methodologies for attachment of prion protein to magnetic nanoparticles can also contribute to enhancing *in vivo *and *in vitro *manipulation of the molecules which is essential in elucidating the structure of the protein and the resulting activity.

**Table 1:** Rate constants of huPrPrec binding onto magnetic and gold coated magnetic nanoparticles.

huPrPrec (μg/ml)	Fe_3_O_4_@Au		Fe_3_O_4_-LAA	
	Rate constant (h^-1^)	R^2^	Rate constant (h^-1^)	R^2^
1	0.06	0.98	0.058	0.94
1.5	0.078	0.97	0.079	0.98
2	0.083	0.94	0.081	0.98
2.25	0.112	0.88	0.113	0.90
3.75	0.191	0.99	0.190	0.98
5	0.194	0.98	0.212	0.99

**Table 2:** Percentage of bonded prion after 20 h reaction time.

huPrPrec (μg/ml)	Fe_3_O_4_@Au	Fe_3_O_4_-LAA
	%	%
1	93	94
1.5	87	87
2	65	67
2.25	70	73
3.75	79	81
5	73	75

## Conclusion

We have demonstrated the possibility of immobilizing huPrPrec onto magnetic and gold coated magnetic nanoparticles by functionalization of these particles with appropriate chemistry to bear carboxyl groups. The immobilization methodologies developed in this study and the information on prion binding kinetics will be useful for sensitive and label-free detection of prion proteins, and will be helpful in the assessment of the physiological and pathological condition of these proteins. We also envision that the immobilization methodologies discussed could be applied for rapid identification, epidemiological studies, genetic evaluation, and forensic investigation.

## Methods

### Preparation of magnetic nanoparticles

Iron (II) chloride tetrahydrate 97 %, iron (III) chloride hexahydrate 99%, sodium hydroxide, acetic anhydride, nitric acid, 1-butanol, octane, toluene, methanol, L-aspartic acid (LAA), cetyltrimethylammonium bromide (CTAB), 3-mercaptopropionic acid (3-MPA), sodium tetrahydridoborate (NaBH_4_), and Phosphate Buffer Saline (PBS), pH 7.4 were obtained from Sigma-Aldrich Inc. (St Louis, USA). Hydrogen tetrachloroaurate (III) hydrate (HAuCl_4_) was obtained from Sigma-Aldrich Inc. (Milwaukee, WI). Streptavidin, a strain of *Streptomyces *avidini was purchased from Sigma-Aldrich Inc. (MO, USA) and 1-ethyl 3-(3-dimethylaminepropyl) carbodiimide hydrochloride (EDC) from Pierce (Rockford, IL, USA) was used to complete the streptavidin-biotin reaction in the presence of *N*-hydroxysuccinimide (NHS) (Sigma-Aldrich, Allentown, PA). Human recombinant prion protein histidine-tagged (23–231), huPrPrec was obtained from Abcam Inc (Cambridge, MA).

### Preparation of Fe_3_O_4 _magnetic nanoparticles

Magnetic nanoparticles Fe_3_O_4 _were prepared by hydrothermal co-prepcipitation of ferric and ferrous using NaOH as a base as described by Kouassi et al [21]. Typically iron (II) chloride and iron (III) chloride (1:2) were dissolved in nanopure water at a concentration of 0.25 M iron ions and chemically precipitated at room temperature (25°C) by repeatedly adding 1 M NaOH to maintain a constant pH of 10. The precipitates were heated at 80°C for 35 min under continuous mixing and washed 4 times in water and several times in ethanol. During washing, the magnetic nanoparticles were separated from the washing liquid using a magnetic separator of strength greater than 20 megaoersted (MOe). The particles were finally dried in a vacuum oven at 70°C.

### Synthesis of gold-coated magnetic nanoparticles (Fe_3_O_4_@Au)

Fe_3_O_4_@Au were prepared using reverse micelle of CTAB using 1-butanol as a cosurfactant and octane as the oil phase by modification of the procedure developed by Jun *et al*. [22]. The size of the reverse micelle is dependent on the molar ratio of water to surfactant. In this work, particles were prepared by choosing a molar ratio of water to CTAB, *w *as [H20]/[CTAB] = 8. The procedure and components of the experiment were described by Jun *et al*. [19]. To a 2.5 ml of solution A containing 1 M FeCl_3_, 0.5 M FeCl_2_, 0.17 mole of CTAB, 0.7 mole of butanol, and 0.17 mole of octane, was added a 2.5 ml of solution B containing 1 M NaBH_4 _and the same composition of CTAB, butanol, and octane as in solution A. The blend was heated at 60°C while vigorously mixing for 20 min to form magnetic nanoparticles. A 2 ml amount of a solution C containing 0.44 mole of HAuCl_4_, 0.8 mole of CTAB, 0.25 mole butanol, and 0.011 mole octane and an equivalent volume of a solution D containing 1.6 M NaBH_4_, 0.8 mole of CTAB, 0.25 mole of butanol, and 0.11 mole of octane were successively added. The pH was kept at 11 by adding minute amounts of 0.5 M NaOH. The mixture was continuously mixed for 15 minutes. The gold coated magnetic nanoparticles formed were washed four times with water, several times with methanol and dried in a vacuum oven for 6 h. To demonstrate that a gold layer was formed around the magnetic nanoparticles, gold colloidal and Fe_3_O_4_@Au solutions were prepared by dissolving 1.5 mg of HAuCl_4 _and Fe_3_O_4_@Au, respectively, in 4 ml of water and the absorbance was measured using a UV-Vis Beckman Du spectrophotometer.

### LAA functionalization of magnetic nanoparticles

1.5 g of magnetic nanoparticles was immersed in 50 ml of 0.1 M LAA solution prepared in nitric acid of pH ≈ 2. The mixture was sonicated for 15 min and vigorously stirred for 10 h at room temperature. An external magnetic field was applied to recover the particles and washed two times with nanopure water. The process is expected to ensure the chemisorption of aspartic acid, bearing carboxyl and amino groups onto the surface of the particles.

### Carboxylation of gold coated magnetic nanoparticles Fe_3_O_4_@Au

The carboxylation of gold coated magnetic nanoparticles was done to allow the formation of amide bond between carboxyl groups on the surfaces of the nanoparticles with amino groups from the protein molecules. Magnetic nanoparticles (1.5 g) were added to 15 ml ethanolic solution of 3-MPA 20 mM, sonicated for 48 h and rinsed in nanopure water and dried in a vacuum oven for 6 h.

### Immobilization of prion protein (huPrPrec) onto magnetic and gold coated magnetic nanoparticles

One mg of EDC, 1.2 mg of NHS and 8 mg of carboxyl or LAA functionalized particles were added to 3 ml phosphate buffer solution (pH 7.4) containing 3–15 μg of huPrPrec. The mixture was then sonicated at 4°C for 10 min and continuously shaken for 18 h at room temperature. At 4 h time intervals a magnetic separator was used to separate the particles from the reaction medium and 30 μl of the reacting solution was taken and used for the determination of protein content using the Bio-Rad Protein Assay using human IGg as the protein standard. Portion of the particles were taken and washed with PBS to separate unbound protein from the particle surfaces and used for characterization.

### Binding kinetics

The concentration of protein in the supernatant was monitored every four hours for a period of twenty hours to evaluate the concentration of bound huPrPrec and the rate constants were determined using linear regression analysis from the plot of protein concentration *versus *time. Each point is the average of two measurements. The sensitivity of the assay was demonstrated by examining the dose response of the rate constant versus huPrPrec concentration in the concentration range between 2 and 5 μg/ml.

### Characterization

The sizes of magnetic and gold coated magnetic nanoparticles were characterized by transmission electron microscopy (TEM, JEM 1200 EXII, JEOL) and the attachment of biomolecules qualitatively by FTIR spectroscopy (Biorad FTS 6000, Cambridge, MA). The samples for TEM analysis were prepared as follows: a drop of magnetic nanoparticles was dispersed in nanopure water and the resulting solution was sonicated for 5 min to obtain better particle dispersion characteristics. A drop of the dispersed solution was then deposited onto a copper grid and dried overnight at room temperature. Confirmation of the binding of huPrPrec onto the magnetic nanoparticles was done using FTIR spectroscopy. Nanoparticles bearing huPrPrec obtained after the immobilization procedure were separated using a magnetic separator and washed with PBS to remove unbound huPrPrec. A small amount of the remaining huPrPrec-magnetic nanoparticle conjugates were mixed in 5 ml of PBS and a drop of the mixture was deposited on the FTIR micro-ATR sample holder for analysis.

## Authors' contributions

Authors Kouassi and Irudayaraj were both responsible for the concept, the planning of the experiments, the data analysis, and the writing of manuscript.
